# Permanent Neonatal Diabetes Caused by Creation of an Ectopic Splice Site within the *INS* Gene

**DOI:** 10.1371/journal.pone.0029205

**Published:** 2012-01-03

**Authors:** Intza Garin, Guiomar Perez de Nanclares, Elena Gastaldo, Lorna W. Harries, Oscar Rubio-Cabezas, Luis Castaño

**Affiliations:** 1 Endocrinology and Diabetes Research Group, Hospital de Cruces, Universitat Politècnica de València/Euskal Herriko Unibertsitateko, Barakaldo, Bizkaia, Spain; 2 Centro de Investingación Biomèdica en Red de Diabetes y Endermedades Metabólicas (CIBERDM), Centro de investigación Biomèdica en Red de Endermedads Raras (CIBERER), Barakaldo, Bizkaia, Spain; 3 Pediatric Endocrinology, Hospital de La Ribera, Alzira, Valencia, Spain; 4 Institute of Biomedical and Clinical Science, Peninsula College of Medicine and Dentistry, Exeter, United Kingdom; 5 Department of Pediatric Endocrinology, Hospital Infantil Universitario Niño Jesús, Madrid, Spain; 6 Centro de Investigación Biomédica en Red Fisiopatogía se la obesidad y Nutrición, Instituto de Salud Carlos III, Madrid, Spain; Institut Jacques Monod, France

## Abstract

**Background:**

The aim of this study was to characterize the genetic etiology in a patient who presented with permanent neonatal diabetes at 2 months of age.

**Methodology/Principal Findings:**

Regulatory elements and coding exons 2 and 3 of the *INS* gene were amplified and sequenced from genomic and complementary DNA samples.

A novel heterozygous *INS* mutation within the terminal intron of the gene was identified in the proband and her affected father. This mutation introduces an ectopic splice site leading to the insertion of 29 nucleotides from the intronic sequence into the mature mRNA, which results in a longer and abnormal transcript.

**Conclusions/Significance:**

This study highlights the importance of routinely sequencing the exon-intron boundaries and the need to carry out additional studies to confirm the pathogenicity of any identified intronic genetic variants.

## Introduction

Neonatal diabetes mellitus is a rare genetic disorder (1∶100,000 live newborns) characterized by hyperglycemia and low insulin levels presenting during the first 6 months of life [1]. The disease can be clinically subdivided into transient (TNDM) and permanent (PNDM) forms depending on whether or not insulin dependence resolves in infancy.

In the last few years, a number of mutations in the preproinsulin gene (*INS*) (summarized by Stoy et al) [2] have been found to be the second most common cause of PNDM (12% according to Edghill et al. and 24.3% according to Colombo et al.) [3], [4].

Initially, heterozygous coding mutations were identified [1], [3]–[7]. These mutations show a dominant inheritance and most of them have been found to disrupt the folding and conformation of proinsulin *in vitro*, thus inducing endoplasmatic reticulum (ER) stress [4]. Clinical features of these patients suggest that birth weight is similar [3] or slightly higher [4] compared to birth weight in patients with mutations in *KCNJ11* and *ABCC8*, although patients with heterozygous mutations in the *INS* gene tend to be diagnosed slightly later [2]–[4].

Subsequently, recessively-acting biallelic *INS* mutations were described which result in decreased insulin biosynthesis through distinct mechanisms, including gene deletion, abnormal transcription, lack of the translation initiation signal, and altered mRNA stability. Compared to patients carrying heterozygous *INS* mutations, patients with biallelic *INS* mutations show a markedly different clinical phenotype, with lower birth weight (median: 1680 g vs 2530 g) and an earlier age at diagnosis (median: 1 week vs 10 weeks) [8], [9].

We report the identification of a PNDM patient carrying a heterozygous mutation within the terminal intron of the *INS* gene. The mutation results in aberrant splicing by introducing an ectopic splice site leading to the insertion of 29 nucleotides from the intron into the mature mRNA and therefore resulting in a longer and abnormal transcript.

## Results and Discussion

Since the first description of *INS* as a PNDM gene in 2007, several dominantly-acting mutations (23 missense, one nonsense, one insertion/deletion) [2] and a further ten recessively-acting mutations have been reported to cause neonatal diabetes [8]. They are located in all regions of the preproinsulin gene, including the promoter (6 mutations), signal peptide (3 mutations), B-chain (12 mutations), C-peptide (2 mutations), A-chain (8 mutations), and both pairs of basic amino acids flanking the C-peptide (2 mutations) [2]. *In vitro* studies for some of these mutations have confirmed [4], [10], [11] or discarded [12] their pathogenic effect, thus highlighting the importance *in vitro* studies for the functional characterization of genetic variants identified in rare diseases, where familial co-segregation studies cannot usually be performed since there is often one affected individual at each family.

On the other side, no intronic *INS* mutation affecting splicing has been described to date. The majority of splicing-disrupting mutations detected in other genes are single nucleotide substitutions within the consensus sequence of the splice sites and result in either complete exon skipping, use of a nearby pseudo 3′ or 5′ splice site, or retention of the mutated intron [13]. Less frequently, the mutations create an ectopic splice site or activate a cryptic splice site, thereby changing the overall splicing pattern of the mutant transcript [14].

**Figure 1:**
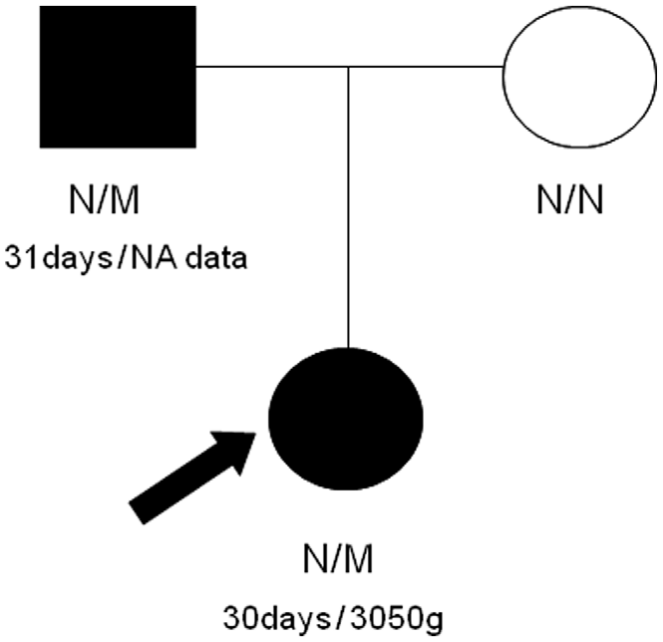
Partial pedigree of the family with intronic INS mutation. (N, Normal allele; M, mutation). Solid black-filled shapes represent patients with permanent neonatal diabetes. Age at diagnosis and birth weight are shown below the symbols (NA: not available).

**Figure 2:**
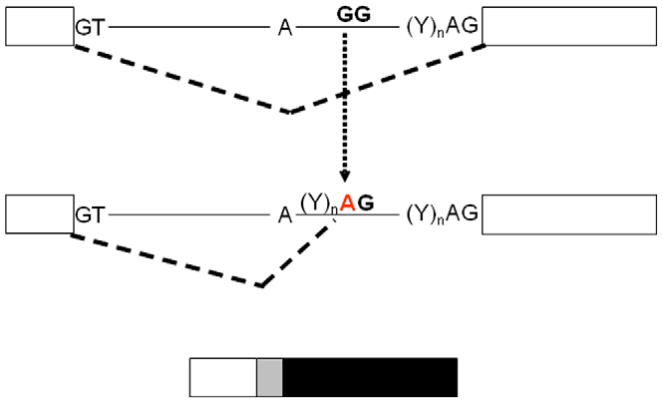
Schematic representation of the intron-exon structure of the INS gene and the splicing changes due to the found mutation. Exons are represented by white boxes, introns as lanes, the intronic insertion at transcript level is indicated by a grey box and the new aberrant sequence is shown in black. The splicing event is shown by a broken lane. The mutation position is indicated in red. Y: C/T.

In the present study, we have identified the first heterozygous intronic mutation (c.188-31G>A) in the *INS* gene causing neonatal diabetes in both the proband and her affected father (Figure 1). The variant is located 31 bp proximal to exon 3 within the polypyrimidine tract of intron 2, which normally determines the strength of the splice acceptor site. The mutation turns the dinucleotide (Y)_n_ **G**G into (Y)_n_ **A**G, creating a typical consensus 3′ splice site, as *in silico* analysis predicted. Indeed, the confidence value for the new ectopic splice site was higher than that for the regular splice site (0.52 vs 0.18) so that the splicing machinery would preferentially recognise the new splice site and read the last 29 bp of intron 2 as exonic sequence (Figure 2).

**Figure 3:**
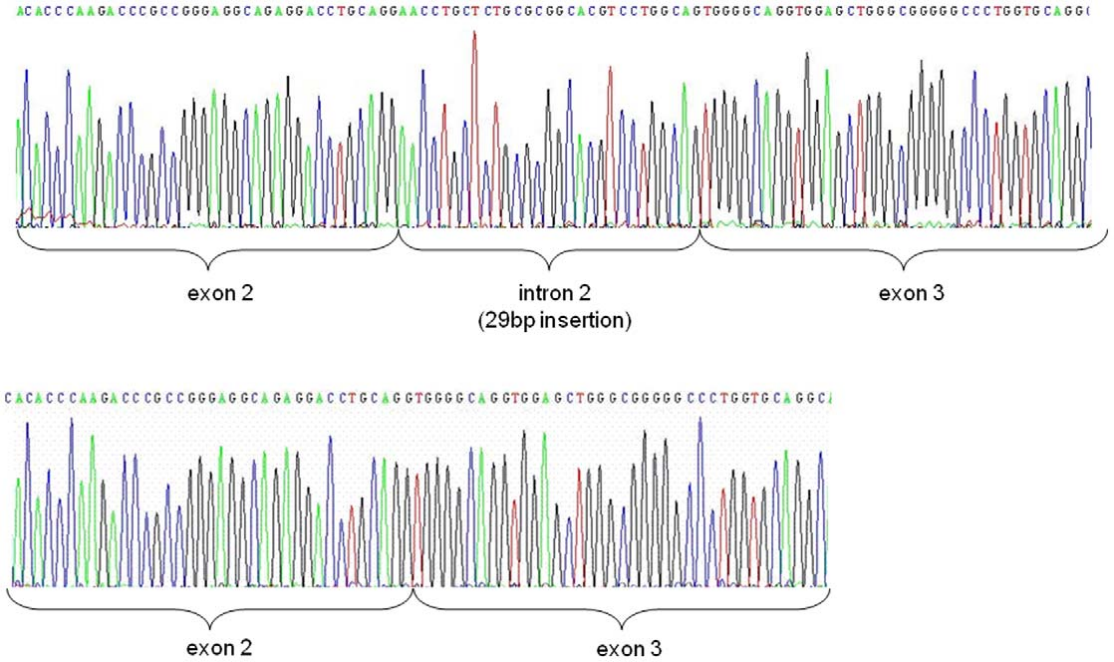
Sequence of amplified cDNA obtained from RNA of lymphoblastoid cells. Upper sequence is obtained from patient's immortalized cells. Lower panel, the sequence obtained from unaffected control lymphoblastoid cells.

**Figure 4:**
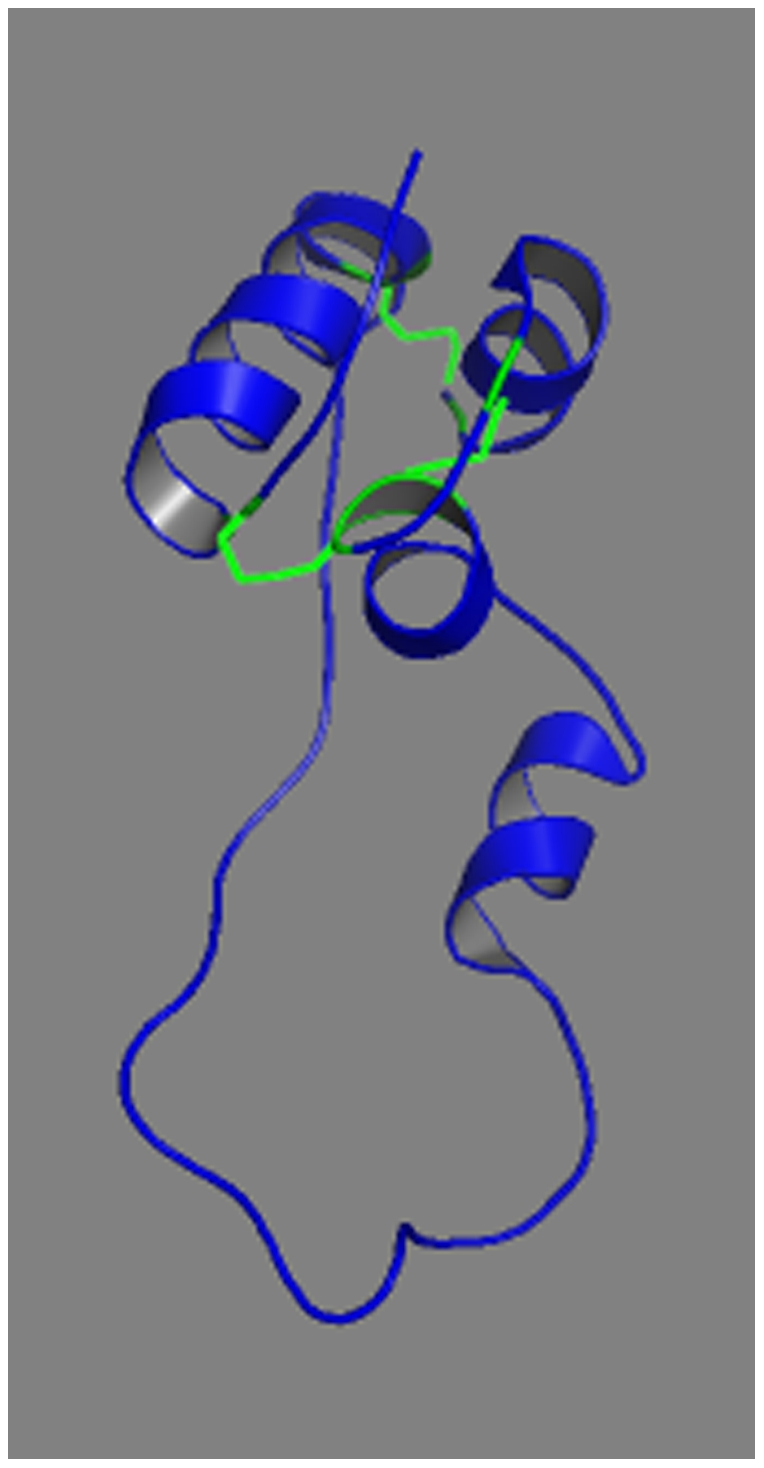
Tertiary structure of the wild-type proinsulin (PDB ref: 2KQP). Disulfide bonds (B7-A7, B19-A20, A6-A11) are shown in green.

**Figure 5:**
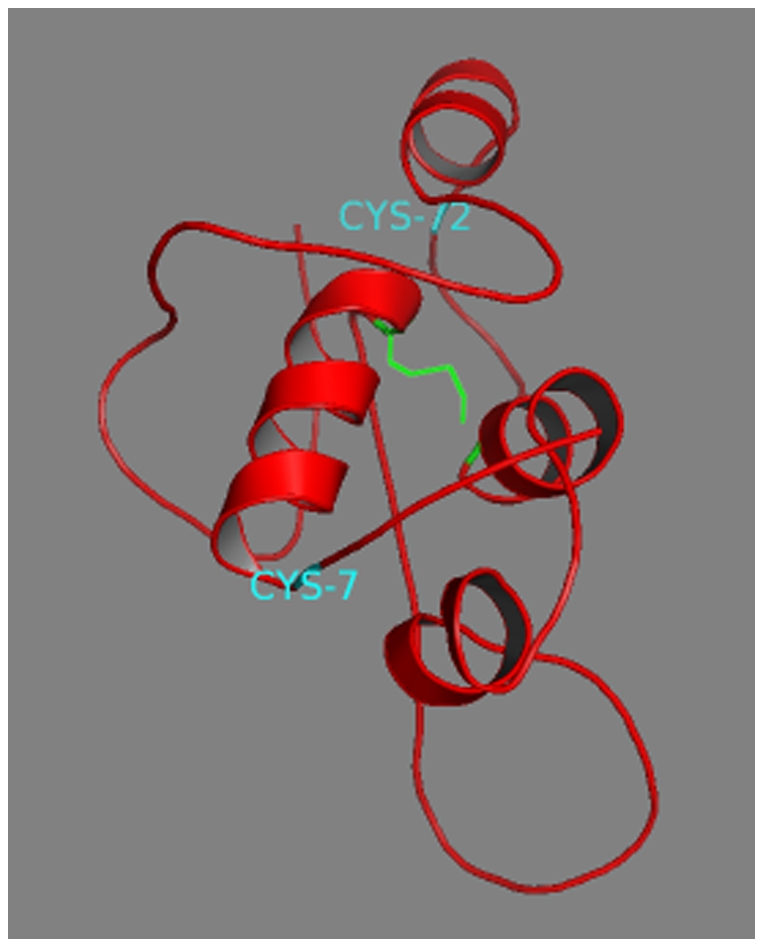
Predicted tertiary structure of the aberrant protein. The new disulfide bond (B19-N25) is shown in green. Unpaired cysteine residues (B7 and N34) are shown in cyan. N25 and N34: cysteine at position 25 and 34 of the “new” aminoacidic sequence. N34 correspond to Cys72.

**Figure 6:**
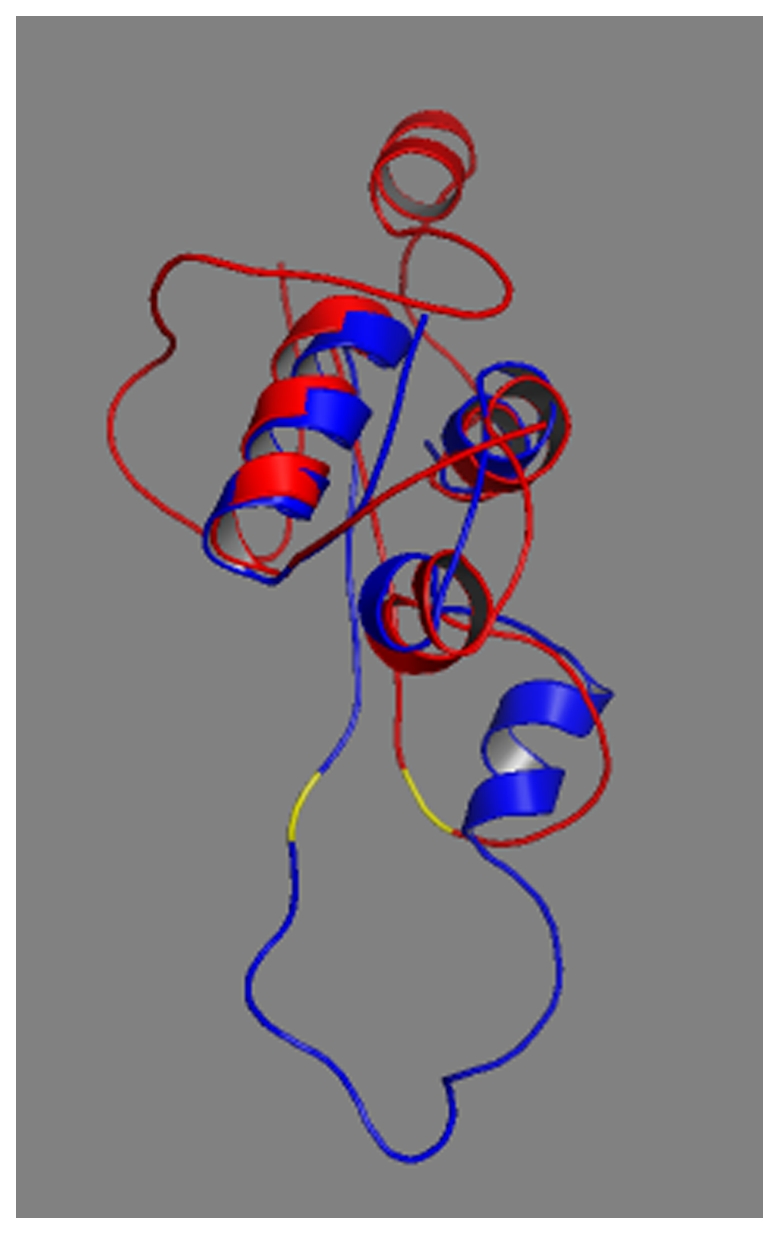
Superposition of both proteins. Arg31,32 cleavage site is shown in yellow in both the normal and mutant chains.

RNA studies carried out on a lymphoblastoid cell line obtained from the proband confirmed this hypothesis (Figure 3). The 29 bp insertion alters the reading frame in the mutant transcript which is not predicted to be subject to nonsense-mediated [15] nor non-stop decay [16] since the frame shift would not generate a premature termination codon and the new stop codon would be located within the 3′ UTR (19 aminoacids downstream the original stop codon). In fact, according to the intensity of the bands obtained after RT-PCR (results not shown), the mutant transcript was processed more efficiently than the normal transcript, as predicted by *in silico* studies. The mutant proprotein would normally translocate into the ER as the signal peptide is intact. *In silico* 3D structure studies of its amino acid sequence (p.Val63Glufs*78) predicted the removal of the three conserved disulfide bonds (B7-A7, B19-A20, A6-A11) and the creation of a new one (B19-N25), suggesting that mutant protein would fail to fold properly (Figures 4 and 5). However, the B-chain would be normal and Arg31,32 cleavage site remains accessible (Figure 6).

Although it has not yet been established clearly the sequence of events leading to initiation of B-chain folding, formation of inter- and intrachain disulfide bond and proinsulin cleavage, misfolding would disrupt trafficking from the endoplasmic reticulum which would ultimately lead to β-cell dysfunction and eventual cell death [17], as it happens with a number of other previously described heterozygous *INS* mutations [2].

Clinically, the birth weight of the proband was normal. Although birth weight in patients carrying *INS* heterozygous coding mutations tend to be low [2], some cases with normal birth weight have been reported [4]. It has been proposed that the growth retardation at birth might be partly explained by the gender of the patient, being more severe in males than in females [18]. In our experience, however, low birth weight is usually associated with recessively-inherited *INS* mutations that lead to a nearly complete insulin deficiency [8], whereas heterozygous mutations leading to a gradual and progressive decrease of insulin secretion secondary to ER stress-mediated β-cell apoptosis are associated with normal or slightly reduced birth weight [3]–[5]. The proband was diagnosed at 5 weeks of age, slightly earlier that the reported median age of 10 weeks [2], [3], but this may reflect an increased awareness due to the positive family history.

In summary, we report a novel mutation type at the preproinsulin gene leading to neonatal diabetes. Our finding highlights the importance of routinely sequencing exon-intron boundaries and the need to carry out additional RNA studies to confirm the pathogenicity of any identified intronic genetic variants.

## Materials and Methods

### Ethics statement

The study was carried out in accordance with the Declaration of Helsinki. This study was approved by the Medical Ethics Commission, Hospital de Cruces. The study also complied with Spanish laws and regulations, accreditation standards and institutional policies. Participants gave written informed consent, with parents consenting on behalf of the proband.

### Case report

The proband, of Caucasian origin, presented with permanent diabetes at 2 months of age (blood glucose 8.3–10 mmol/L). She was born to non-consanguineous parents at 40 weeks gestation with a normal birth weight (3050 g, −0.66 SDS). Pregnancy was uneventful. Pancreatic autoantibodies (ICA and IAA) were absent at diagnosis and she was started on subcutaneous insulin (initial daily requirement 0.4 U/kg). There was a strong family history of early-onset diabetes on the paternal side. Her father was diagnosed with permanent neonatal diabetes (PNDM) at 31 days of life and two paternal aunts died from diabetic ketoacidosis within the first year. The proband is currently 10.5 years and remains on intensive insulin therapy (1.1 U/kg/day). Diabetes has never remitted.

### Molecular genetic analysis

Genomic DNA from proband and parents was extracted from peripheral leukocytes using standard procedures using QIAamp DNA Mini Kit (QIAGEN, Düren, Germany). Regulatory elements and coding exons 2 and 3 of the *INS* gene were amplified and sequenced as previously described [8]. Additionally, 100 unrelated, non-diabetic individuals were studied, and served as control group.

### In silico studies

The potential effect on mRNA splicing of the c.188-31G>A mutation was analyzed using the splice site prediction of *CBS-Gene Finding and Intron Splice Site Prediction* (http://www.cbs.dtu.dk/services/NetGene2/) web interface.

### RNA analysis

Cell lines were established from peripheral blood lymphocytes derived from the proband and an unaffected control by EBV transformation. Cell lines were maintained in 1× RPMI-1640 (Gibco Life Technologies, Paisley, UK), supplemented with 10% fetal calf serum (Gibco Life Technologies, Paisley, UK). Total RNA was extracted from approximately 1×10^6^ EBV-transformed lymphoblastoid cellsusing QiaAmp Blood RNA mini kit (QIAGEN, Düren, Germany). Synthesis of complementary DNA (cDNA) and amplification of the fragment of interest were performed from mRNA using the One Step RT-PCR (QIAGEN, Düren, Germany) (primers and conditions are available on request). RT-PCR products were run on 4% agarose gels and the amplicons were excised and purified by QIAquick Gel Extraction Kit (QIAGEN; Düren, Germany). The purified amplicons were sequenced by standard methods on an ABI3130XL (Applied Biosystems, Warrington, UK).

### Protein Structure Prediction

3D structure of the mutant protein was predicted using I-TASSER fold recognition method [19]. The predicted structure was superimposed with the normal proinsulin structure (PDB ref: 2KQP) using TM-Align [20] and visualised with PyMOL Software (www.pymol.org).
